# Outcome Goals and Health Care Preferences of Older Adults With Multiple Chronic Conditions

**DOI:** 10.1001/jamanetworkopen.2021.1271

**Published:** 2021-03-24

**Authors:** Mary E. Tinetti, Darcé M. Costello, Aanand D. Naik, Claire Davenport, Kizzy Hernandez-Bigos, Julia R. Van Liew, Jessica Esterson, Eliza Kiwak, Lilian Dindo

**Affiliations:** 1 Department of Internal Medicine, Yale School of Medicine, New Haven, Connecticut; 2 Department of Chronic Disease Epidemiology, Yale School of Public Health, New Haven, Connecticut; 3 Center for Innovations in Quality, Effectiveness and Safety (CIN 13-413), Michael E. DeBakey Veterans Affairs Medical Center, Houston, Texas; 4 Houston Center for Innovations in Quality, Effectiveness, and Safety (IQuESt), Baylor College of Medicine, Houston, Texas; 5 Brookdale Department of Geriatrics and Palliative Medicine, Icahn School of Medicine at Mount Sinai, New York, New York; 6 Department of Behavioral Medicine, Medical Humanities, and Bioethics, Des Moines University, Des Moines, Iowa

## Abstract

**Importance:**

Older adults with multiple chronic conditions (MCCs) vary in their health outcome goals and the health care that they prefer to receive to achieve these goals.

**Objective:**

To describe the outcome goals and health care preferences of this population with MCCs.

**Design, Setting, and Participants:**

This cross-sectional study included participants in the Patient Priorities Care study who underwent health priorities identification from February 1, 2017, to August 31, 2018, in a primary care practice. Patients eligible to participate were 65 years or older, English speaking, and had at least 3 chronic conditions; in addition, they used at least 10 medications, saw at least 2 specialists, or had at least 2 emergency department visits or 1 hospitalization during the past year. Of 236 eligible patients, 163 (69%) agreed to participate in this study. Data were analyzed from August 1 to October 31, 2020.

**Exposures:**

Guided by facilitators, participants identified their core values, as many as 3 actionable and realistic outcome goals, health-related barriers to these goals, and as many as 3 helpful and 3 bothersome health care activities.

**Main Outcomes and Measures:**

Frequencies were ascertained for outcome goals and health care preferences. Preferences included health care activities (medications, health care visits, procedures, diagnostic tests, and self-management) reported as either helpful or bothersome.

**Results:**

Most of the 163 participants were White (158 [96.9%]) and women (109 [66.9%]), with a mean (SD) age of 77.6 (7.6) years. Of 459 goals, the most common encompassed meals and other activities with family and friends (111 [24.2%]), shopping (28 [6.1%]), and exercising (21 [4.6%]). Twenty individuals (12.3%) desired to live independently without specifying necessary activities. Of 312 barriers identified, the most common were pain (128 [41.0%]), fatigue (45 [14.4%]), unsteadiness (42 [13.5%]), and dyspnea (19 [6.1%]). Similar proportions of patients identified at least 1 medication that was helpful (130 [79.8%]) or bothersome (128 [78.5%]). Medications most commonly cited as helpful were pain medications, including nonopiods (36 of 55 users [65.5%]) and opioids (15 of 27 users [55.6%]); sleep medications (27 of 51 users [52.9%]); and respiratory inhalants (19 of 45 [42.2%]). Most often mentioned as bothersome were statins (25 of 97 users [25.8%]) and antidepressants (13 of 40 users [32.5%]). Thirty-two participants (19.6%) reported using too many medications. Health care visits were identified as helpful by 43 participants (26.4%); 15 (9.2%) reported too many visits. Procedures were named helpful by 38 participants (23.3%); 24 (14.7%) cited unwanted procedures. Among 48 participants with diabetes, monitoring of glucose levels was doable for 18 (37.5%) and too bothersome for 9 (18.8%).

**Conclusions and Relevance:**

Participants identified realistic and actionable goals while varying in health care activities deemed helpful or bothersome. The goals and health care preferences of more diverse populations must be explored. Previous work suggests that clinicians can use this information in decision-making.

## Key Points

**Question:**

What are the common goals and health care preferences of older adults with multiple health conditions?

**Findings:**

In this cross-sectional study, 163 older adults reported 459 goals, most commonly encompassing activities with family and friends (24.2%), shopping (6.1%), exercising (4.6%), and living independently (4.4%). Medications most commonly cited as helpful were nonopioid pain medications (65.5% of users), sleep medications (52.9% of users), and inhalants (42.2% of users), whereas statins (25.8% of users) and antidepressants (32.5% of users) were the most commonly reported bothersome medications.

**Meaning:**

Participants identified realistic and doable goals and health care preferences; this information can inform decision-making.

## Teaser

This cross-sectional study describes the outcome goals and health care preferences of older adults with multiple chronic conditions.

## Introduction

Clinical decision-making can be difficult for the two-thirds of older adults who have multiple chronic conditions (MCCs).^1^ These individuals are excluded from many clinical trials and may accrue less benefit or greater harm than suggested by disease guidelines because of coexisting conditions.^2,3,4,5,6^ Most treatments are recommended to address disease-specific outcomes or survival, whereas older adults with MCCs vary in the outcome goals that they most desire.^7,8,9^ Furthermore, the medications, health care visits, testing, procedures, and self-management tasks entailed in treating MCCs require investments of time and effort that may be burdensome and conflict with what patients are willing and able to do.^10,11,12^

There is growing awareness of the need to transition health care, particularly for persons with MCCs, from treating single diseases in isolation to health care that is aligned with patients’ priorities (ie, values, goals, and preferences).^5,9,13,14,15,16,17^ Most tools available for eliciting patients’ goals and preferences were developed in the context of advanced illness or specific conditions or populations.^18,19,20,21,22,23,24,25,26,27,28,29^ Some of these approaches may be less useful to older adults weighing the benefits, burdens, and tradeoffs associated with long-term management of several chronic conditions. Goal attainment scaling is appropriate for this latter population, although some applications include goal categories representing medical and supportive care, which are better considered care preferences that support attainment of desired life goals.^22,23,24,25,26,27,28,29^

In response to the need for approaches that support the alignment of clinical decision-making with the priorities of older adults with MCCs, a diverse group of patients, health care systems experts, and clinicians developed Patient Priorities Care (PPC).^13,14^ Aimed at both patients and clinicians, PPC begins with a structured process in which patients work with a member of the health care team to identify the outcome goals that they most desire to achieve based on what matters most to them (ie, their values-based outcome goals) and to specify what they are willing (or unwilling) to do to achieve those outcomes (ie, their health care preferences).^30,31,32^ Clinicians then use the results of these facilitated discussions to align their decisions and care with patients’ priorities.^31,32,33,34^

Evidence from a recent study^31^ indicated that the PPC framework was feasible to patients and clinicians and was associated with increased care aligned with patients’ priorities and decreased treatment burden and unwanted health care. The aim of the present study is to describe the outcome goals and health care preferences identified by older adults in this study.^26^

## Methods

### Study Design and Setting

Details of the PPC study have been described elsewhere.^30,31,33^ Briefly, 10 primary care clinicians from a large multisite primary care practice in Connecticut invited their patients to participate in the PPC study during a routine visit. Relevant to the present cross-sectional study, patients who agreed to participate met with 1 of 2 trained members (K.H.-B. and an advanced practice registered nurse) of the practice to identify their priorities. The institutional review board at the Yale University School of Medicine, New Haven, Connecticut, approved this study; oral consent was obtained for all participants. We followed the Strengthening the Reporting of Observational Studies in Epidemiology (STROBE) reporting guidelines for cross-sectional studies.^35^

### Participants

Inclusion criteria consisted of being 65 years or older, the ability to speak English, and having at least 3 chronic conditions; in addition, participants used at least 10 prescription medications, were seen by 2 or more specialists, or had at least 2 emergency department visits or 1 hospitalization during the past year. Exclusion criteria included advanced dementia, hospice eligibility, receiving dialysis, or nursing home residence. Potentially eligible participants were identified through electronic health record data. Of the 236 eligible patients treated by clinicians at the PPC practice, 163 (69.1%) agreed to participate in the PPC study and were included in the present analysis.

### Patient Priorities Identification Process

Development of the process to identify patients’ priorities was described previously.^30^ Details of the identification process are included in the facilitator manual.^36^ To summarize, a priorities facilitator (either an advanced practice registered nurse or a case manager [K.H.-B.] trained to elicit patients’ priorities) met with patients at the practice site, at home, or by telephone from February 1, 2017, to August 31, 2018. Priorities identification required 20 to 30 minutes, usually during a single session. The process began with identifying values, that is, who and what matters most. We started with values because they tend to remain stable, whereas outcome goals often vary with life and health changes.^37,38^ To identify personalized values, patients were asked questions such as “What does enjoying life mean to you?” and “When you have a good day, what happens?” When needed, facilitators used prompts drawn from 4 previously identified values domains, including social connections and relationships, enjoying life and productivity, functioning and independence, and managing health and symptoms.^37,38^ Facilitators then helped patients to identify as many as 3 outcome goals that reflected their values and were specific, actionable, and realistic enough to inform clinical decision-making. To link the outcome goal to their values, facilitators told participants: “Goals should be based on your values, or what’s important to you in life. We’ve talked about what matters most to you. Now let’s talk about how you would show that you are living out those important things. Let’s identify activities that you want to be able to do that reflect what is important to you.” Participants were informed: “A health goal can be something you want to keep doing, or something you would like to be able to do more of. Goals need to be specific and realistic so that your doctor can work with you on making sure your health care is focused on achieving that goal.” Prompts and probes were used to get the goals as specific, actionable, and realistic as possible. Participants then were asked what health problems or symptoms they believed most interfered with or were barriers to their identified outcome goals. Participants next identified as many as 3 health care activities (ie, medications, health care visits, procedures, diagnostic tests, and self-management tasks) that they thought would help most with their outcome goals, were doable, and were not too difficult or bothersome and as many as 3 health care activities in these categories that they found burdensome, bothersome, unhelpful, or that they wanted to stop if possible. Together these were considered health care preferences.

Facilitators recorded the results in a template that included participants’ key values, outcome goals, and any barriers to achieving them and their health care preferences. Completed templates were transmitted to the electronic health record. A sample completed template is in the eFigure in the Supplement.

### Statistical Analysis

Demographic data were ascertained during the baseline telephone interview and verified by electronic health record review. Chronic conditions were determined from the problem list and medications from the medication list of the primary care practice’s electronic health records at the time of study enrollment. Medications included those prescribed for chronic conditions. Medications used for time-limited conditions (eg, antibiotics) were not included.

Participant characteristics were summarized with descriptive statistics. The outcome goals and health care preferences were obtained from the deidentified priorities templates. One author (M.E.T.) initially combined goals that reflected similar activities. The other authors reviewed and reached consensus on the goal groupings listed. The health care preferences were aggregated into the predetermined categories of medications, health care visits, procedures and tests, and self-management tasks. Frequencies were ascertained for the outcome goals and health care preferences.

## Results

Participant baseline characteristics are shown in Table 1. Among the 163 participants, most were White (158 [96.9%]) and women (109 [66.9%]), with 54 men (33.1%) and a mean (SD) age of 77.6 (7.6) years. Of the 140 participants for whom educational attainment was ascertained, 59 (42.1%) reported at least some college. Participants had a median of 4 (interquartile range, 3-5) chronic conditions.

**Table 1.** Characteristics of Study Participants

Characteristic	Patient data (n = 163)^a^
Age, mean (SD), y	77.6 (7.6)
Female sex	109 (66.9)
Male sex	54 (33.1)
White	158 (96.9)
Nonwhite^b^	5 (3.1)
At least some college	59 (36.2)
Health insurance	
Traditional Medicare	74 (45.4)
Medicare Advantage	65 (39.9)
Medicare-Medicaid	24 (14.7)
Chronic conditions^c^	
Arthritis	77 (47.2)
Atrial fibrillation	33 (20.2)
Chronic lung disease	22 (13.5)
Depression	41 (25.2)
Diabetes	48 (29.4)
Heart failure	12 (7.4)
Hypertension	127 (77.9)
No. of chronic conditions, median (IQR)	4 (3-5)
Prescription medications used^d^	
Statin	97 (59.5)
β-blocker	66 (40.5)
Diuretic	47 (28.8)
Other cardiovascular^e^	108 (66.3)
Inhalant	45 (27.6)
Oral hypoglycemic agent^f^	36 (75.0)
Insulin^f^	16 (33.3)
Antidepressant	40 (24.5)
Benzodiazepine	37 (22.7)
Nonbenzodiazepine^g^	14 (8.6)
Opioid	27 (16.6)
Nonopioid pain medication	55 (33.7)
No. of prescription medications, median (IQR)	7 (5-9)

Abbreviation: IQR, interquartile range.

^a^ Unless otherwise indicated, data are expressed as number (percentage of patients).

^b^ The nonwhite group included African Americans.

^c^ Identified from active problem list in the electronic health record at time of study enrollment.

^d^ Identified from prescription medication list in the electronic health record at time of study enrollment.

^e^ Includes other than statins, β-blockers, and diuretics.

^f^ Includes the 48 participants who had a diagnosis of diabetes.

^g^ Includes trazodone hydrochloride (n = 6) and other nonbenzodiazepine sleep medications (n = 8). Nonprescription medications, such as antihistamines and melatonin, were not included.

### Outcome Goals

The 163 participants identified 459 outcome goals (Table 2). Participants’ intent was unclear for an additional 5 responses, exemplified by: “I want to be able to get up in the morning.” The most commonly mentioned outcome goals encompassed activities with family and friends, involving both meals (36 [7.8%]) and other events, such as visiting or playing with grandchildren (75 [16.3%]; total, 111 [24.2%]). Other commonly cited outcome goals included shopping (28 [6.1%]) and exercising as an enjoyable and productive activity (21 [4.6%]).

**Table 2.** Health Outcome Goals Among Older Adults With Multiple Chronic Conditions

Health outcome goal groups	No. (%) of goals (n = 459)^a^	Examples of health outcome goals (barriers)^b^
Participate in activities other than meals with family or friends	75 (16.3)	Visit son at the nursing home weekly Play with great grandchildren when they come over each week (knee pain)
Have meals with family and friends with whom they did not live	36 (7.8)	Cook dinner and host her children each month (none) Go down to the dining room to eat and socialize each day (fatigue) Go out to dinner with family (so many instructions for meal preparation because of diabetes)
Shop	28 (6.1)	Shop once per week with spouse (fatigue) Shop with wife each week (pain, neuropathy, imbalance)
Exercise other than walking (go to gym [n = 7]; golf [n = 5]; swimming [n = 4]; yoga [n = 3]; ballroom dancing [n = 1], and Tai Chi [n = 1])	21 (4.6)	Keep going to pool every day to swim (shortness of breath) Swim with my granddaughter 3 times per week (arthritis pain) Be able to play golf again (muscle pain, neuropathy, imbalance) Go to the gym for zumba and water aerobics (loss of feeling in feet) Continue to do yoga 3-5 times per week (lower back pain)
Stay in own home; take care of self; live independently (no specific, actionable activity mentioned)	20 (4.4)	Continue to live alone in own house (none) Keep caring for self (balance difficulty and lack of sleep) Continue to manage apartment and live independently (none)
Walk as exercise	19 (4.1)	Walk with his wife once per week around the complex (back pain) Will go for a walk around the neighborhood each week with daughter (lack of motivation and knee pain)
Take care of or help family or friends (spouse, children, grandchildren, sibling, mother, friend)	18 (3.9)	Assist wife with care needs daily (none) Babysit grandchildren each day to help daughter while she works (none) Provide daily care to mother (none)
Volunteer (eg, senior center, health setting, gallery, community organizations, teaching)	18 (3.9)	Coordinate charitable activities at church once a week (stress incontinence) Volunteer at community organization once per week (light headedness; muscle cramping and knee pain) Continue to be president of the club (none)
Artistic activities (painting and drawing [n = 6], photography [n = 2], crafts [n = 2]; knitting or crocheting [n = 4]; woodworking [n = 2]; music [n = 1])	17 (3.7)	Continue to do hobbies each week that include photography, making miniatures, drawing, and painting (none) Make crafts and sell them to have spending money (hand pain) Would like to do drawing and ceramics again (arthritis pain) Continue to knit (thumb pain)
Take distant trips	16 (3.5)	Fly each week to be with wife at second home (none) Go to favorite place with daughter 3 times per year (back pain) Continue to travel abroad, next year going to Southern Europe (dizziness)
Go to religious service or participate in church activities	15 (3.3)	Go to church each week (difficulty walking and imbalance) Go Bible study each week (lack of sleep) Drive to church each week (weight and shortness of breath)
Participate in organizations or community activities	15 (3.3)	Go the senior center once per week (dizziness)
Participate in leisure and recreational activities (outdoor activities not otherwise named [n = 5]; fish [n = 3]; bowl or play cards [n = 2]; go to beach [n = 1]; camp [n = 1])	13 (2.8)	Continue outdoor activities with sister (bone pain) Go fishing each week (lymphedema and leg wraps) Bowl every Tuesday (burning pain)
Go to the casino	13 (2.8)	Go to the casino each week (lightheadedness)
Garden	12 (2.6)	Do gardening at least once per week (body pain)
Go out to eat (mentioned as the specific desired activity)	12 (2.6)	Go out to lunch without a problem (lunchtime insulin)
Do housekeeping activities	12 (2.6)	Perform a housecleaning task each day (knee pain) Clean own home (muscle cramping)
Cook and bake	11 (2.4)	Cook meals independently (muscle spasms) Can with wife weekly and make sausage or sauerkraut monthly (dizziness and back pain) Cook each day for self and for son (Coumadin [warfarin] diet a barrier)
Drive (mentioned as the goal activity)^c^	10 (2.2)	Continue to be able to drive and go places when wants each week (none) Drive at night (cataracts)
Take local trips	8 (1.7)	Take day trips (fatigue)
Build, maintain, and repair things	8 (1.7)	Do some renovations around the house (pain) Continue to renovate home, install sheet rock, and paint (muscle pain and burning in legs)
Personal mobility including standing, walking, transferring, climbing stairs	8 (1.7)	Be able to walk and climb stairs more easily to be more active with wife (poor sleep, fatigue, pannus) Be able to stand long and steady enough to put things into a drawer (imbalance)
Attend performances (sport events [n = 3]; theater [n = 2]; music [n = 2])	7 (1.5)	Continue to drive to theater 4 times per year with friends (none)
Do paid work (eg, CNA; retail store; freelance writer; appliance repair)	7 (1.5)	Work at least once per week as a CNA (none) Perform parttime job of 4 hours per week at store (back pain) Continue working on appliances and run business (hand pain)
Care for a pet	6 (1.3)	Walk dog around the complex twice a day to see people (weight) Take care of 3 cats (none)
Do yard and lawn work	6 (1.3)	Continue to mow lawn (shortness of breath)
Go to the hairdresser (n = 4) or barber (n = 1)	5 (1.1)	Drive to the hairdresser each week (urinary urgency)
Do cognitive leisure activities (computer [n = 3]; puzzles [n = 1]; reading [n = 1])	5 (1.1)	Do puzzles each day (none) Do something on the computer daily (grogginess)
Shop as a hobby (antiques, tag sales, flea market)	5 (1.1)	Go shopping for antiques each month (lack of energy)
Connect with family or friends but no specific activity mentioned	3 (0.7)	Maintain connection with son who lives in a different state (none)
Manage symptoms, health problems (muscle strength, pain)^d^	3 (0.7)	Be as healthy as possible when grandchild is born (back pain)
Do something enjoyable or meaningful but no specific activity mentioned	2 (0.4)	Be able to enjoy retirement with wife someday (none)
Get out and be independent but no specific activity mentioned	2 (0.4)	Get out and be independent (pain)
Dress independently	1 (0.2)	Keep being able to get up and get dressed (back pain)
Live longer for an important event	1 (0.2)	Wants to see granddaughter expected in several months (disease progression)

Abbreviation: CNA, certified nursing assistant.

^a^ The 163 participants could identify as many as 3 health outcome goals; of the 464 responses, 459 health outcome goals were identified; 5 responses could not be categorized.

^b^ Responses participants gave when asked what health problems, symptoms, or health care they believed most interfered with or were barriers to their identified outcome goals are in parentheses.

^c^ Driving was mentioned by other participants but in the context of accomplishing another health outcome goal.

^d^ Although only 4 participants identified managing a symptom or health problem as their primary health outcome goal, all participants were asked about and most could identify a specific symptom, health problem, or health care task that was a barrier to achieving their primary health outcome goals.

Goals could be specific, such as “I want to go down to the dining room to eat and socialize each day.” In other cases, they were less specific, such as, “I want to help my sister every day.” Twenty individuals (12.3% of participants and 4.4% of goals) noted the desire to live independently or stay in their home but did not identify specific activities needed to ensure this could happen. Some outcome goals expressed a desire to continue a current activity (eg, “I want to continue to babysit my grandchildren every day”), whereas others reflected the aspiration to do something they were unable to do for health reasons, such as “I would like to be able to play golf again.”

Many health goals addressed more than 1 value. Most often this involved a function, such as personal mobility, cooking, driving, or traveling, that supported a goal related to relationships, enjoying life, or productivity (eg, “I want to cook and host my children for dinner each month” or “I want to continue to drive to the opera house 4 times a year with my friends”).

### Barriers to Achieving Outcome Goals

Although only 4 individuals reported outcome goals related to managing health or living longer—all of whom linked their health to meaningful events—participants did identify a health-related barrier to 312 of 459 outcome goals (68.0%). The most commonly mentioned barriers were pain (128 [41.0%]); fatigue, lack of energy, or poor sleep (45 [14.4%]); gait imbalance, unsteadiness, or neuropathy (42 [13.5%]); and shortness of breath and dizziness (19 [6.1%] each). Some participants identified multiple barriers to their goal.

### Health Care Preferences

Health care activities that participants perceived as helpful and doable are shown in Table 3, whereas those activites that they believed were burdensome or bothersome are listed in Table 4. Similar numbers of participants mentioned at least 1 medication that was helpful and acceptabale (130 [79.8%]) and at least 1 medication that was bothersome or unhelpful (128 [78.5%]). Thirty-two participants (19.6%) believed they were taking too many medications; 57 (35.0%) reported having bothersome symptoms from their medications but did not mention specific medications (Table 4).

**Table 3.** Health Care Activities Older Adults With Multiple Chronic Conditions Believed Were Helpful and Acceptable

Health care activity	No. (%) of participants (n = 163)^a^	Examples of participant comments
**General medication-related**		
Able to manage medications	10 (6.1)	Uses written log with check boxes for medication administration Does not mind taking all the medications Has a system: “They are in this shoebox; does the set up each week.”
Mentioned ≥1 medication that was helpful or tolerable	130 (79.8)	Thinks most of the medications are doing their job and “I don’t have muscle pain and my bladder and bowels are good”
**Specific medications**		
Prescription nonopioid pain medications^b^	36 (65.5)	Diclofenac gel is helping joint pain “Gabapentin helps so much, I would get an electric shock pain before” Glucosamine 3 times a day helps
Opioids^c^	15 (55.5)	Hydrocodone worked great for the leg jumping and to help sleep Tramadol has been handling the pain Naproxen was better, “but I understand why he switched me and tramadol helps my back pain”
Benzodiazepine or nonbenzodiazepine^d^	27 (52.9)	Alprazolam helps a great deal Diphenhyrdramine helps sleep and stops the worry and over thinking
Inhalants^e^	19 (42.2)	Inhalers help with shortness of breath
Antidepressants^f^	7 (17.5)	SSRI is helping “My depression medications are working and I don’t want the depression to get worse”
Statins^g^	5 (5.2)	Takes cholesterol medications because “the older you get, the more issues you will have if your heart is bad”
β-blockers and other cardiovascular medications^h^	13 (8.0)	Blood pressure medications are not causing dizziness Atenolol is great: “it helps my a-fib” “Very happy with blood pressure pills” and “my pressure is perfect” “I have a-fib and the enlarged heart, so I know why I have to take the heart medications”
Insulin^i^	6 (37.5)	“The APRN at my endocrinologist’s office recently put me on Tresiba [insulin degludec] and my blood sugars have really improved” Used to insulin, has no problem “injecting myself”
Other medications mentioned as helpful^j^	30 (18.4)	Furosemide is not causing bothersome frequent urination Omeprazole is helping, “I have never felt this good”; Toviaz works for the ride so they do not have to stop
**Health care visits**		
Clinician visits (primary care [n = 8]; medical specialists [n = 10] including cardiology, pulmonary, oncology, gastrointestinal tract, neurology)	18 (11.0)	Sees Dr X (primary care) and Dr Y (cardiology); “they handle things” “My pulmonologist saved my life”
Rehabilitation (physical therapy [n = 12]; cardiac rehabilitation [n = 1], pulmonary rehabilitation [n = 1])	14 (8.6)	Physical therapy helped with shoulder pain Physical therapy helping with recovery from knee surgery Pulmonary rehabilitation helped a great deal
Other health care visits (pain management [n = 3]; ophthalmology [n = 3]; urology [n = 2]; wound care center [n = 2]; spine center [n = 1])	11 (6.7)	Requesting referral to pain clinic
**Procedures and tests**		
Helpful or desired procedures (eye [n = 5]; knee [n = 3]; urinary incontinence [n = 2]; gastrointestinal tract [n = 2]; pacemaker and defibrillator [n = 2]; cardiac [n = 1]; back [n = 1]; lung reduction [n = 1]; cochlear implant [n = 1]; not stated [n = 1])	19 (11.7)	Very pleased with recent knee surgery The heart operation helped with fatigue and shortness of breath Cataract surgery was helpful Will consider knee surgery if recommended Would like to try hearing implant surgery
Diagnostic tests (INR [n = 11]; pacemaker checks [n = 3]; bone density [n = 3]; diagnostic imaging [n = 2]; preoperative tests [n = 1])	20 (12.3)	Willing to do INR draws despite stating that they are a “pain in the neck” Would prefer at-home blood draws Willing to perform tests to be cleared for surgery
**Self-management tasks**		
Diet (diabetes [n = 7]; weight loss [n = 3]; high fiber [n = 1]; renal [n = 1]; multiple [n = 1]; other or not specified [n = 9])	22 (13.5)	Willing to see nutritionist for diabetes management Manages 4 different diets; it is hard but is “happy to do it to help with avoiding diabetes, reflux, my stomach issues, and diverticulitis”
Assistive device (walker [n = 8]; cane [n = 8]; leg, knee, or back brace [n = 6]; wheelchair [n = 1]; chair lift [n = 1]; stair rail [n = 1])	25 (15.3)	Uses cane “every time I walk; I need it because of the hip” Must use the walker or knees give out Knee brace helps with the pain shooting down from back
Glucose level monitoring^k^	18 (37.5)	Blood glucose level check 1-3 times per week Blood glucose level check every other day: “every day is too much” Checks blood glucose levels once in a while
CPAP	14 (8.6)	“I know that if I don’t use my CPAP, I see the immediate effects and a big difference in my energy” “My CPAP is very helpful; I don’t wake up with headaches anymore”
Hearing aids	3 (1.8)	Pleased with new (fifth) set of hearing aids
Blood pressure and weight monitoring	3 (1.8)	Checks blood pressure at home
Other self-management (compression stocking [n = 5]; leg wrap [n = 3]; eye drops [n = 2]; ostomy [n = 1]; self-catheterization [n = 1])	12 (7.4)	Willing to wear the wraps every day to avoid the wound center Administering eye drops without a problem

Abbreviations: a-fib, atrial fibrillation; APRN, advanced practice registered nurse; CPAP, continuous positive airway pressure; INR, international normalized ratio; NSAID, nonsteroidal anti-inflammatory drug; SSRI, selective serotonin reuptake inhibitor.

^a^ The 163 participants were able to identify as many as 3 health care activities they considered helpful and acceptable. Percentages calculated based on 163 participants unless otherwise stated.

^b^ Includes the 55 participants who received prescription nonopioid pain medications.

^c^ Includes the 27 participants who received opioid medications.

^d^ Includes the 51 participants who received either a benzodiazepine (n = 37) or a prescription nonbenzodiazepine sleep medication, either trazodone hydrochloride (n = 6) or another nonbenzodiazepine sleep medication (n = 8); nonprescription sleep medications such as antihistamines and melatonin were not included.

^e^ Includes the 45 participants who received inhalants or nebulized medications.

^f^ Includes the 40 participants who received antidepressants.

^g^ Includes the 97 participants who received statins.

^h^ All 163 participants used to calculate denominator because most received at least 1, and other than a single mention of atenolol, participants did not name specific medications but rather the conditions for which medications were prescribed (eg, blood pressure, atrial fibrillation). Seven participants mentioned that blood pressure medications were not causing adverse effects; 6 mentioned a benefit such as blood pressure or heart rate control.

^i^ Includes the 16 participants who received insulin.

^j^ Included docusate (Colace), donepezil hydrochloride, eyedrops, fesoterodine fumarate, furosemide, histamine 2-blocker, “medication for restless leg syndrome,” meclizine hydrochloride, metformin hydrochloride, mirabegron, polyethylene glycol (Miralax), muscle relaxers, nitroglycerin patch, omeprazole, rituximab, tamoxifen citrate, tamsulosin hydrochloride, combined carbidopa-levodopa (Sinemet), warfarin, and rivaroxaban (Xarelto).

^k^ Includes the 48 participants who had a diagnosis of diabetes.

**Table 4.** Health Care Activities Older Adults With Multiple Conditions Believed Were Burdensome, Bothersome, Unhelpful, or Unwanted

Health care activity	No. (%) of participants (n = 163)^a^	Examples of participant comment
**General medication-related**		
Too many medications	32 (19.6)	“This pile of meds is not an appealing breakfast” Too many prescribed medications are not helpful Wants to keep decreasing the number of medications
Mentioned ≥1 medication that was bothersome	128 (78.5)	Getting weak and shaky: “I don’t know if I'm taking too much of something”
Mentioned adverse effects of medications; no specific medication mentioned^b^	57 (35.0)	Wants to know if medications could be adding to muscle pain Does not want any more medications that can cause dizziness: “That is how I fell” Losing weight, not sure if it is the medications Constant stomachache; unsure if medications are contributing Gets dry mouth from all the medications
**Specific medications**		
Statin^c^	25 (25.8)	“I have certain sensations when I take the statin, sometimes leg cramps” Atorvastatin calcium led to muscle pain Legs feel heavy in the morning, does not know if that is because of the new cholesterol medication Recently decreased simvastatin dose, muscles were aching “so bad and now they are okay”
β-blockers^d^	9 (13.6)	Dizzy since prescribed carvedilol, 25 mg twice daily “Atenolol, 50 mg, was making me feel so off, I was afraid to leave my home, I was so dizzy and thought I might be going crazy” Cutting back on the metoprolol has helped feel less tired.
Other cardiovascular medications^e^	11 (10.2)	In the past using 3 blood pressure medications, “it was making me feel so off, I was afraid to leave my home, I was so dizzy and thought I might be going crazy”
Antidepressants^f^	13 (32.5)	“Is this combination of medications along with my depression medications causing this cloudy thinking?” Remeron caused agitation Tried the citalopram hydrobromide for 6 days and stopped it “because of a feeling of hot saliva, like blood, that I was swallowing”
Opioids^g^	10 (37.0)	“Tramadol knocks me out, I don’t like that” “Pain pills make me constipated” “Oxycontin [oxycodone hydrochloride] made me climb the walls”
Nonopioid pain medications^h^	9 (16.4)	Takes Lyrica (pregabalin) at night because “it makes me dizzy” Gabapentin is not helping “as much as I hoped” “I can’t have NSAIDs because of my stomach bleeds”
Insulin^i^	9 (56.3)	Insulin administration 4 times per day is bothersome “Insulin is bothersome, why can’t I just be on the pills if my numbers are good” “I want to continue to be a caregiver for my daughter, sliding-scale insulin make this difficult and burdensome”
Benzodiazepine or nonbenzodiazepine^j^	8 (15.7)	Will not take a prescription medication for sleep because it will “knock me out for 2 days” “The sleeping pills make me too groggy in the morning” “I stopped clonazepam because of my breathing, it caused the need for the CPAP”
Diuretics^k^	7 (14.9)	Furosemide is helping but “I am peeing so much” “They want me to take the water pill, I am going all afternoon and all night”
Other medications mentioned as burdensome^l^	18 (11.0)	“I can’t take PO meds for my osteoporosis because they caused chest pain”
**Health care visits**		
Too many clinician visits or specialists	15 (9.2)	Would like less frequent clinician visits Wants to limit number of specialists seen “I’m tired of going to so many doctors” “I know that my care is specialized but I want 1 person to look over everything because all the specialists tell me different things”
Rehabilitation (physical therapy; pain management)	8 (4.9)	Did not do the physical therapy this round, “I don’t think it helped” The exercises put pressure on right shoulder Went to pain management; the injections did not work
Counselling	3 (1.8)	“I have seen 3 therapists in the past, I don’t think I should see a fourth”
**Procedures and tests**		
Unwanted procedures (most commonly mentioned unwanted procedures: knee [n = 7]; shoulder /rotator cuff [n = 5])	24 (14.7)	“I don’t think I want the operation for my knees even if I have to live with this pain” “I won’t have the knee surgery because I am too worried about anesthesia and I don’t know how it will affect my mind and my memory” Does not want bowel/colorectal surgery Does not want back surgery
Diagnostic tests (diagnostic imaging [n = 1]; blood tests [n = 1]; mammogram [n = 1]; Papanicolaou smear [n = 1]; memory evaluation [n = 1]; colonoscopy [n = 1])	6 (3.7)	“I know they want me to go for the scans and the tests but I’m not going through that” “I really want to talk about my blood, to see if I don’t have to go that blood work anymore” Mammogram and Papanicolaou smears: “I am trying to cut back on things because I am 90”
**Self-management tasks**		
Glucose level monitoring^m^	9 (18.8)	“I don’t check my blood sugar because I hate needles” “I don’t check my blood glucose, I didn’t like the new meter”
CPAP	9 (5.5)	Cannnot tolerate the CPAP “I am using the CPAP and it is not helpful, I still feel exhausted in the mornings, it is not helpful and very bothersome”
Diet	6 (3.7)	Seeing the nutrition person was not helpful; “she told me to add up all the grams for every single thing that I buy, I can’t do that, that is too much, I feel like she didn’t help me” Not motivated to diet or make healthier food choices at this time
Assistive device	6 (3.7)	Quad cane catches left foot “I won’t use a cane because that is embarrassing” “The walker helps me but it doesn’t let me go into places and be safe”
Hearing aids	5 (3.1)	Bilateral hearing aids, I need them but sometimes they pop out” “I finally got new hearing aids, when I closed the fridge it sounded like a bomb went off”
Other self-management tasks	4 (2.5)	“I want to continue to be a caregiver for my daughter each day. Health care tasks make this difficult, burdensome”

Abbreviations: CPAP, continuous positive airway pressure; PO, oral.

^a^ The 163 participants were able to identify as many as 3 health care activities they considered burdensome, unhelpful, or unwanted.

^b^ The most commonly mentioned adverse effects were dizziness or unsteadiness (n = 18), muscle pain (n = 11), gastrointestinal tract complaint (n = 7), dry mouth (n = 4), fatigue (n = 4), and weight change (n = 6).

^c^ Includes the 97 participants who received statins.

^d^ Includes the 66 participants who received β-blockers.

^e^ Includes the 108 participants who received cardiovascular medications other than statins or β-blockers (ie, calcium channel blockers, angiotensin-converting enzyme inhibitors, angiotensin II receptor blockers, other antihypertensives, antiarrhythmics, and long-acting antianginals).

^f^ Includes the 40 participants who received antidepressants.

^g^ Includes the 27 participants who received opioid medications.

^h^ Includes the 55 participants who received prescription nonopioid pain medications.

^i^ Includes the 16 participants who received insulin.

^j^ Includes the 51 participants who received either a benzodiazepine (n = 37) or a prescription nonbenzodiazepine sleep medication, either trazodone hydrochloride (n = 6) or another nonbenzodiazepine sleep medication (n = 8); nonprescription sleep medications such as antihistamines and melatonin were not included.

^k^ Includes the 47 participants prescribed a diuretic.

^l^ Other medications mentioned as burdensome included anticoagulants, bisphosphonates, mirabegron, oral hypoglycemic agents, oxybutynin, prednisone, ropinirole hydrochloride, solifenacin succinate, and tamsulosin hydrochloride.

^m^ Includes the 48 participants who had a diagnosis of diabetes.

Medications used to treat pain were mentioned most commonly as helpful, including nonopioids (36 of 55 users [65.5%]) and opioids (15 of 27 users [55.6%]) (Table 3). Also commonly cited as helpful were benzodiazepine and nonbenzodiazepine medications for sleep (27 of 51 users [52.9%]) and inhalants for respiratory symptoms (19 of 45 [42.2%]). The percentages of medication users mentioning bothersome effects were 9 of 55 users of nonopioids (16.4%), 10 of 27 users of opioids (37.0%), and 8 of 51 users of sleep medications (15.7%); no users mentioned bothersome effects for inhalants (Table 4).

Five of 97 participants receiving statins (5.2%) mentioned benefits or lack of adverse effects (Table 3), whereas 25 (25.8%) ascribed bothersome effects to the medication (Table 4). A high proportion of recipients also reported bothersome effects for antidepressants (13 of 40 users [32.5%]) and insulin (9 of 16 users [56.3%]) (Table 4).

Primary and specialty care, rehabilitation, and other health care visits were identified as helpful by 43 participants (26.4%): “My pulmonologist saved my life” and “Physical therapy helped with shoulder pain” (Table 3). Fifteen participants (9.2%) reported having too many visits or clinicians: “I’m tired of going to so many doctors” (Table 4).

Procedures and tests were mentioned as acceptable and helpful by 38 participants (23.3%). They mentioned both past procedures (“I’m very pleased with my recent knee surgery”) and ones they hoped to have (“I would like to try hearing implant surgery”) (Table 3). Twenty-four participants (14.7%) cited proposed surgical procedures they did not want, with orthopedic procedures mentioned most commonly (12 [7.4%]) (Table 4).

Diet was the most commonly mentioned helpful self-management task (20 [12.3%]) (Table 3); only 6 participants (3.7%) reported diets as burdensome or unwanted (Table 4). Among the 48 patients with diabetes, monitoring of glucose levels was acceptable for 18 (37.5%) (Table 3), although not as frequently as prescribed (“I do my blood sugar check every other day, every day is too much”), and too bothersome to 9 others (18.8%) (Table 4). Other commonly cited activities included continuous positive airway pressure, which was helpful to 14 and bothersome to 9 participants and assistive devices (helpful to 21 and bothersome to 6 participants). The total numbers of participants using continuous positive airway pressure or assistive devices were unavailable.

## Discussion

Identifying outcome goals that are realistic and actionable appears to be feasible among older adults with MCCs. No participant-selected goals were grandiose or unrealistic, likely because arriving at realistic and actionable goals given each person’s health status was part of the facilitation process. Goals often were linked to multiple values as expected, and reported previously, for meaningful human activities.^39^ Functional activities, such as driving or cooking, were often mentioned as ways to connect to people, enjoy life, or be productive. Knowing the values underlying goals can be helpful in patient-clinician communication and decision-making. For instance, if a patient’s outcome goals are not achievable or realistic given their health status, a conversation might include, “I worry that you might not be able to continue driving your friends to the theater. I wonder if there are other ways to fulfill your desire to see shows and connect with your friends that could be more achievable.”^34^

Although few outcome goals focused solely on managing health, most participants did cite health problems, symptoms, or impairments that interfered with their outcome goals. These findings suggest that managing health might best be considered as a way to help achieve life goals for persons with MCCs. Only 1 participant mentioned living longer. Perhaps more deliberative probing might have revealed how participants valued quality vs quantity of life, which was how the value of managing health was originally framed.^37,38^ Alternatively, this tradeoff may have less meaning for people not facing life-threatening situations.^40^

Approaches to identifying patients’ goals have been reported for specific health problems or patient populations.^18,19,20,21,22,23,24,25,26,27,28,29^ A goal taxonomy recently was developed for persons with functional limitations and complex care needs.^26,27^ The patient priorities identification process builds on these efforts, particularly goal attainment scaling, by implementing a process that facilitates the separate identification of value-based outcome goals and health care preferences for persons with MCCs.^30^ This separation supports clinical decision-making by focusing decision-making on the achievement of each person’s outcome goals within the context of the health care the individual is willing and able to receive.

Participants varied in their health care preferences, the aspects of their current health care that they believed were acceptable and helpful, and the aspects that were burdensome, bothersome, or unhelpful. Although instructed to define helpful care in relation to their outcome goals, persons usually mentioned treatments that were helpful for individual conditions, such as hypertension or diabetes.

Not surprisingly, medications were the most frequently cited health care activity. Participants mentioned preventive medications, particularly statins, more often in the context of burden than benefit. Whether they were correct in ascribing bothersome effects to these medications is uncertain, but many participants found these medications to be burdensome.^41,42^ This finding is consistent with previous work showing that older adults are reticent to take preventive medications associated with adverse symptoms that they consider to be health outcomes that need to be balanced against future benefit.^43^ By comparison, medications that address current symptoms, such as pain or sleep, were more likely to be perceived as helpful than bothersome, although clinicians and regulators try to limit use of these medications because of adverse effects.^44^ The differing perceptions of some patients and clinicians on the benefits and harms of preventive medications vs medications that alleviate symptoms needs to be acknowledged. Decision-making guided by patients’ outcome goals may help address these discordant perspectives.

A quarter of participants mentioned that visits or clinicians were helpful; 9.2% reported desiring fewer visits and clinicians. Because participants were not required to comment, results do not imply level of satisfaction with visits or clinicians. Some participants wished that 1 person could oversee their care or expressed frustration when clinicians’ recommendations conflicted. Aligning decisions with patients’ priorities is an effective strategy for coordinating care and avoiding conflicting recommendations.^33,34^

Participants typically believed that the procedures they received were helpful. The 15% who raised concerns did so in the context of prospective procedures. More in-depth discussions are necessary to explore individuals’ desires about future procedures. Having gone through the process of identifying their priorities may help guide these discussions, particularly if the tradeoffs are framed within patients’ priorities.

As for other health care activities, individuals varied in their acceptance of self-management tasks, such as monitoring of glucose levels and use of continuous positive airway pressure, diets, assistive devices, and hearing aids. Of note, even among the 37.5% of patients with diabetes who reported that monitoring glucose levels was acceptable, most acknowledged that they did not check their glucose levels as often as recommended.

### Limitations

Participants were drawn from a single practice with a homogeneous patient population; results may not generalize to other populations. Identifying the priorities of diverse groups is essential. It remains to be determined whether clinicians can use patients’ goals to guide decision-making; preliminary evidence suggests they can.^31,32^ Future work requiring larger samples from diverse populations includes categorizing goals based on agreed upon characteristics and ascertaining the abilities required to achieve specific goals, and conversely, the conditions, symptoms, and impairments impeding their achievement. Ultimately, methods will be needed for determining which interventions most effectively support achievement of specific goals.

## Conclusions

Although further research is needed, this study suggests that asking people about their goals and preferences and getting responses that can inform decision-making is feasible. Combining patients’ health conditions, function, and health trajectory with these goals and preferences should focus care for older adults with MCCs. Because various combinations of conditions and impairments as well as social determinants may affect goal achievement, aligning care with patients’ priorities will require input from many health care professionals as well as community and other services. The variability in goals and preferences supports patients’ priorities as the targets toward which to aim all health and support services.

In related work, strategies for aligning decision-making with patients’ outcome goals and health care preferences have been developed and tested.^33,34^ Tips and scripts for addressing challenges, such as nonactionable or nonspecific goals or goals that are not achievable because of health and functional status or because patients are unwilling to receive the health care necessary to achieve their goals, have also been created.^34^ The eventual objective of this line of investigation is an approach to decision-making and care that helps older adults with MCCs achieve what matters most to them while minimizing harm and treatment burden.

## Notes

Supplement. **eFigure.** Patient Priorities Care Health Priorities Template (Facilitated Version)
