# Supplementary material for: Outcome Goals and Health Care Preferences of Older Adults With Multiple Chronic Conditions
Source: JAMA Netw Open. 2021 Mar 24;4(3):e211271. doi: 10.1001/jamanetworkopen.2021.1271 (PMC7991967; doi:10.1001/jamanetworkopen.2021.1271)
Supplement: Supplement. — eFigure. Patient Priorities Care Health Priorities Template (Facilitated Version) [file jamanetwopen-e211271-s001.pdf]

Supplementary Online Content

Tinetti ME, Costello DM, Naik AD, et al. Outcome goals and health care preferences of older adults with multiple chronic conditions. *JAMA Netw Open*. 2021;4(3):e211271. doi:10.1001/jamanetworkopen.2021.1271

eFigure. Patient Priorities Care Health Priorities Template (Facilitated Version)

This supplementary material has been provided by the authors to give readers additional information about their work.

© 2021 Tinetti ME et al. *JAMA Network Open*.

eFigure. Patient Priorities Care Health Priorities Template (Facilitated Version)

What Matters Most (Values): *Doing activities with family and friends*

Most Important Health Goals: Health goals are specific and realistic activities or outcomes that show you are doing what matters most in your life. These health goals are what you want to achieve with your healthcare.

1. *He wants to go out to eat with his wife twice a month*

2. *He wants to get to playing poker with his friends weekly*

Most Bothersome Symptoms or Problems interfering with your health goals:

1. *Feeling tired/lacking energy*

2. *Having urinary incontinence*

Health care preferences (Helpful and burdensome care and medications)

Helpful care: self-management tasks, clinical visits, tests, or procedures, that you think are helping most with your health goals and you can do them without too much difficulty

1. *Visiting primary care clinician*

2. *Doing rehabilitation*

Helpful medications: Medications you think are helping most with your health goals and you can take without too much difficulty

1. *Taking blood pressure meds*

2. *using arthritis cream*

Burdensome care: self-management tasks, clinical visits, tests, or procedures that don't think are helping your goals and are burdensome or too difficult. You should talk with your doctor about whether these are helping your goals. If not, can you stop them or cut back? If they are helping, is there a way to make them less burdensome or less difficult?

1. *Having surgeries – doesn't know how much they help*

2. *Following a special diet – he can't eat what he likes*

Burdensome medications: Medications you don't think are helping your goals and are too burdensome. You should talk with your doctor about whether these are helping your goals. If not, can you stop or decrease? If they are helping, is there a way to make them less burdensome?

1. *Taking water pill – he doesn't like how it makes him go to the bathroom so often*

2. *Injecting insulin – he doesn't like needles, it's hard to measure with his eyesight, and he worries about low blood sugar*

One Thing: Your most important health goal is **(***insert most important health goal***).** From among the *symptoms or health problems, burdensome health tasks or medications, fill in the* ***ONE THING*** *you most want* to focus on so that you can do (*insert most important health goal*) more often or more easily.

The one thing he wants to focus on is feeling less tired so he can go out to eat with his wife and play poker with his friends more often or more easily.

© 2021 Tinetti ME et al. *JAMA Network Open*.
